# Semi-Automated Plaque Assessment in Cardiac CT: Prognostic Value in Long-Term Follow-Up of Intermediate Stenosis

**DOI:** 10.3390/diagnostics16040600

**Published:** 2026-02-18

**Authors:** Laura Zajančkauskienė, Kristina Balnė, Eglė Montrimavičienė, Antanas Jankauskas, Gintarė Šakalytė

**Affiliations:** 1 Department of Radiology, Medical Academy, Lithuanian University of Health Sciences, LT-50161 Kaunas, Lithuania; antanas.jankauskas@lsmuni.lt; 2 Department of Cardiology, Medical Academy, Lithuanian University of Health Sciences, LT-50161 Kaunas, Lithuania; kristina.balne@stud.lsmu.lt (K.B.); egle.montrimaviciene@stud.lsmu.lt (E.M.); gintare.sakalyte@lsmuni.lt (G.Š.); 3 Institute of Cardiology, Medical Academy, Lithuanian University of Health Sciences, LT-50166 Kaunas, Lithuania

**Keywords:** coronary CT angiography, cardiac CT, intermediate stenosis, quantitative plaque assessment, coronary atherosclerotic plaque, high-risk plaque features

## Abstract

**Background/Objectives**: Intermediate coronary artery stenosis is difficult to risk-stratify, as stenosis severity alone often fails to predict events. This study aimed to evaluate whether quantitative CCTA-derived plaque characteristics and lesion morphology are associated with MACE during long-term follow-up. **Methods**: In this single-center prospective study, 128 patients with stable angina symptoms underwent standardized CCTA and were diagnosed with at least one intermediate coronary stenosis (50–69%, CAD-RADS 3). Quantitative parameters of lesion morphology, lumen geometry, vessel wall dimensions, and plaque composition were assessed using semi-automated CCTA adapted plaque analysis (QAngio CT). Patients were followed for a median of 72 months. MACE was defined as a composite outcome of all-cause mortality, target lesion revascularization, non-fatal MI, and stroke. **Results**: During follow-up, 26.6% of patients experienced MACE. High-risk plaque features were more frequent in patients with MACE. Lesions associated with MACE demonstrated significantly smaller lumen area, reduced mean lumen diameter, and decreased vessel wall area at the obstruction site. In addition, plaques leading to adverse events exhibited larger necrotic core areas. Although no single quantitative parameter independently predicted MACE, a combined multivariable model incorporating lumen geometry and plaque composition showed significant prognostic value. **Conclusions**: In patients with intermediate coronary stenosis, lesion-specific quantitative CCTA parameters—particularly luminal geometry and necrotic core extent—provide prognostic information beyond traditional plaque burden and stenosis assessment. Incorporating detailed plaque morphology into routine CCTA evaluation may improve long-term risk stratification and support more individualized clinical management.

## 1. Introduction

Because atherosclerotic changes in chronic coronary artery disease (CAD) develop progressively over many years, long-term follow-up (FU) provides the strongest foundation for reliable prognostic evaluation [1,2,3]. Growing evidence indicates that specific plaque features—such as low-attenuation (necrotic) core, positive remodeling, napkin-ring sign, and total plaque volume—predict myocardial infarction (MI) and other major adverse cardiovascular events (MACE) independently of luminal stenosis severity. Coronary computed tomography angiography (CCTA) is a robust non-invasive modality for assessing both luminal narrowing and plaque composition [4,5,6,7].

The multicenter SCOT-HEART analysis highlighted the prognostic importance of plaque composition beyond stenosis grade [4]. Serial CCTA studies further demonstrate that a higher baseline burden of cardiovascular risk factors accelerates the progression of adverse plaque phenotypes [7] and that quantification of total plaque volume offers superior 10-year risk stratification compared with stenosis assessment alone [6]. Taken together, these data support the concept that long-term, plaque-focused imaging follow-up yields more accurate and clinically meaningful prognostic information than short observation periods.

Despite consensus statements and expert reviews recommending the incorporation of standardized plaque quantification into routine risk assessment, relatively few studies combine comprehensive quantitative plaque analysis with long-term follow-up [8,9,10,11,12]. Most quantitative CCTA studies report follow-up durations of approximately three years, limiting understanding of the long-term prognostic impact of specific plaque phenotypes and the natural evolution of chronic CAD. To address this gap, the present study applied a standardized, semi-automated plaque analysis protocol to evaluate the association between MACE and quantitative CCTA-derived plaque parameters over a 6-year follow-up period.

Intermediate coronary artery (CA) stenosis remains challenging to manage, even when functional testing is negative or inconclusive. In the literature, an intermediate lesion is typically defined as a visually estimated angiographic stenosis of 30–70% [7]. The Coronary Artery Disease Reporting and Data System (CAD-RADS) provides a standardized framework for reporting CAD severity [13], and its reproducibility has been validated across multiple studies, with high inter-observer agreement [14]. To enhance clinical utility and reduce interpretive variability, we restricted the definition of intermediate lesions to CAD-RADS 3 (50–69% stenosis). Semi-automated CCTA-based plaque analysis was performed using QAngio CT (Research Edition) to enable detailed morphological characterization and identification of MACE predictors.

## 2. Materials and Methods

### 2.1. Study Population

A single-center, non-randomized controlled trial was performed at the Departments of Cardiology and Radiology, Lithuanian University of Health Sciences Kaunas Clinics (Kaunas, Lithuania). The study population consisted of 157 consecutive patients with a low to intermediate pretest probability of obstructive CAD, as determined by the Diamond–Forrester model [15]. All participants underwent clinically indicated CCTA between 1 January 2017 and 1 January 2020, and were diagnosed with one or more intermediate CA stenoses. The inclusion criteria were as follows: age > 30 years, stable angina symptoms, no prior history of CAD, preserved left ventricular ejection fraction (LVEF > 50%) assessed by two-dimensional echocardiography, and adequate image quality on CCTA for diagnostic interpretation. Patients with a prior diagnosis of CAD, confirmed either visually or clinically, were excluded from the study. A total of 133 patients met all eligibility criteria and were subsequently included in the final analysis. During the six-year FU period, five patients were lost to FU. In total, 194 coronary stenoses were evaluated. An intermediate lesion was defined as an atherosclerotic plaque producing 50–69% luminal narrowing in a vessel with a reference diameter greater than 1.5 mm, consistent with the CAD-RADS 3 category of the standardized CCTA reporting system. Patients with more severe stenoses were excluded in advance. All participants were prospectively followed for six years from the date of their baseline CCTA, with the observation period concluding on 1 January 2025 (in an earlier publication intermediate results were published after a 2-year FU period [16]). Clinical and demographic data were obtained from the national electronic health record system (eSveikata) and the hospital electronic medical record system of Hospital of Lithuanian University of Health Sciences Kaunas Clinics by dedicated investigators. FU information was obtained through structured telephone interviews and comprehensive review of medical records to identify MACE. Subsequently, patients were stratified into two groups based on event occurrence: those who experienced MACE and those who did not during the FU.

Approval for the study was obtained from Kaunas Regional Biomedical Research Ethics Committee (project identification code BE-2-93, 14 October 2019) and the study was conducted in accordance with the criteria described in the declaration of Helsinki. Written informed consent was obtained from all participants.

### 2.2. Image Acquisition

Baseline CCTA examinations were performed using a 320-detector row CT scanner (Aquilion ONE; Toshiba Medical Systems, Nasu, Japan). Scanning parameters included a detector collimation of 320 mm × 0.5 mm, tube current of 300–580 mA, tube voltage of 100–120 kV, gantry rotation time of 350 ms, and a resulting temporal resolution of 175 ms. A prospective electrocardiogram (ECG)-gated acquisition protocol covering 70–80% of the R–R interval was applied. In patients with a heart rate (HR) ≤ 65 beats per minute, image acquisition was completed within a single cardiac cycle using one-segment reconstruction. For patients with HR > 65 beats per minute, multi-segment reconstruction was performed by combining data from two consecutive cardiac cycles, yielding an effective temporal resolution of 87 ms.

If the HR exceeded 60 beats per minute, beta-blockers were administered as premedication to achieve optimal heart rate control. To improve coronary vasodilation and optimize image quality, sublingual nitroglycerin (0.4 mg) was administered immediately prior to scanning. All participants underwent both non-contrast and contrast-enhanced CT acquisitions. The non-contrast scans were used for CA calcium scoring (Agatston method). The contrast-enhanced CCTA was performed using 70–100 mL of iodinated contrast agent (iopromide, Ultravist 370; Bayer HealthCare, Leverkusen, Germany), with the dose adjusted according to patient body weight. All acquired images were subsequently transferred to and archived in the Picture Archiving and Communication System (PACS) for further analysis.

### 2.3. Imaging Analysis

Qualitative and quantitative plaque morphology assessment, limited to intermediate lesions, was conducted using a dedicated CCTA analysis software (QAngio CT Research Edition, version 2.11.6.1; Medis Medical Imaging Systems, Leiden, The Netherlands). Additionally, routine parameters were evaluated: Agatston score, CT-adaptive Leaman score, Segment Involvement Score (SIS) and high-risk plaque features. Analyses were conducted by two independent, trained observers, in accordance with previously published methodologies [7,17]. CCTA datasets were retrieved from the PACS and imported into the analysis software, which automatically identified the aorta and coronary arteries and generated three-dimensional (3D) reconstructions of the coronary tree. The target vessel and affected segment or branch were then selected for detailed evaluation. Longitudinal views of the selected artery were assessed in multiple imaging planes. The inner (intimal) and the outer (adventitial) vessel contours were automatically delineated and manually adjusted when necessary. The proximal and the distal reference segments were automatically defined and refined manually as needed. After confirmation of vessel boundaries, automated quantification of luminal geometry, vessel dimensions, and plaque characteristics was performed at both the lesion level and the site of maximal luminal narrowing.

### 2.4. Reproducibility of Quantative Plaque Measurements

Bland–Altman analysis demonstrated good intra-observer agreement across quantitative plaque measurements, with parameter-specific mean biases ranging from −0.12% to 0.48% and corresponding 95% limits of agreement between −5.9% and 6.4%. Intra-observer reliability assessed using intra-class correlation coefficients (ICC) (two-way random-effects model, absolute agreement) indicated good repeatability, with ICC values ranging from 0.75 to 0.88. Inter-observer reproducibility also demonstrated good agreement, with mean biases ranging from 0.8% to 3.2% and 95% limits of agreement between −7.4% and 9.1%. Corresponding ICC values for inter-observer comparisons ranged from 0.70 to 0.85, consistent with good absolute agreement.

### 2.5. Study Endpoints

The primary study endpoint was the occurrence of MACE. MACE was defined as a composite outcome of all-cause mortality, target lesion revascularization, non-fatal MI—as specified by the European Society of Cardiology criteria [18]—and stroke (defined as neurologic symptoms associated with radiologic findings). In instances where multiple adverse events occurred simultaneously or sequentially, they were recorded as a single composite event, with the time-to-event calculated from the date of enrollment to the first recorded event.

### 2.6. Statistical Analysis

Statistical analyses were performed using IBM SPSS Statistics for Windows, version 20.0 (IBM Corp., Armonk, NY, USA). A *p* value < 0.05 was considered statistically significant.

Both descriptive and inferential statistical methods were applied. Categorical variables were expressed as frequencies and percentages, whereas continuous variables were reported as mean ± standard deviation (SD) or median with interquartile range (IQR), depending on data distribution. Baseline demographic and plaque-related characteristics were compared between patients who experienced MACE and those who did not. The Wilcoxon–Mann–Whitney and Kruskal–Wallis tests were used for comparisons of continuous variables, and the Chi-square (χ^2^) test was applied for categorical variables. Variables showing statistically significant differences between groups were subsequently incuded in univariate and multivariate Cox proportional hazards regression models to assess their independent prognostic value for MACE occurrence.

## 3. Results

### 3.1. Baseline Characteristic

More than half of the studied popuation were women (*n* = 69; 53.9%). The mean age of the study cohort was 66.3 ± 10.0 years. Demographic and clinical parameters of the MACE and non-MACE groups are presented in Table 1.

In the MACE group, the number of men and women was equal (*n* = 17; 50%, same in both groups), whereas in the non-MACE group women were more prevalent (*n* = 52; 55.3%). The mean age was 67.8 ± 10.4 years in the MACE group and 66.4 ± 9.4 years in the non-MACE group (*p* = 0.909). Thus, no significant differences in age or sex were observed between the groups. Although the majority of the study population has shown a high cardiovascular risk profile, including hypertension (84.4%, *p* = 0.086) and dyslipidemia (74.2%, *p* = 0.419), both groups were homogeneous regarding CAD risk factors, medications and laboratory tests, with no statistically significant differences between them (the most clinically important presented in Table 1).

**Figure 1:**
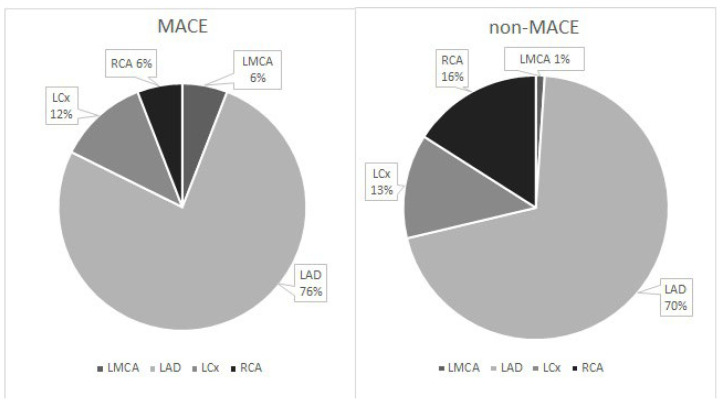
Intermediate CA stenosis localization in MACE and non-MACE group. LMCA—left main coronary artery; LAD—left anterior descending coronary artery; LCx—left circumflex coronary artery; RCA—right coronary artery.

The distribution of intermediate CA stenosis localization did not differ significantly between groups (*p* = 0.209). In both groups, the most common location was the left anterior descending artery (LAD) (MACE—76%; non-MACE—70%) (Figure 1).

The median FU duration was 72.4 (67.2; 78.4) months, and the median time-to-event was 557.0 (134.0; 1834.0) days. The total MACE rate over the 6-year FU was 34 people (26.6%). The shortest time to event was 21 days, whereas the longest was 2567 days. Target lesion revascularization was the most frequent MACE (in all cases percutaneous coronary intervention was performed) (*n* = 26; 76.5%), followed by non-fatal MI (*n* = 4; 11.8%), all-cause death (*n* = 3; 8.8%) and stroke (*n* = 1; 2.9%).

### 3.2. Quantitative Evaluation of Coronary Atherosclerotic Burden

#### 3.2.1. Routine Evaluation

First, the CA atherosclerotic burden was assessed using scoring systems commonly applied in clinical practice. The Agatston score tended to be higher in the MACE group compared to the non-MACE group (130.5 (50.0; 363.0) and 93.0 (23.5; 248.5) respectively, *p* = 0.136). The CT-adaptive Leaman score and the SIS showed only minimal differences between the groups (Table 2). Overall, the coronary atherosclerotic burden assessed by CCTA-derived scoring systems did not differ significantly between patients with and without MACE.

**Figure 2:**
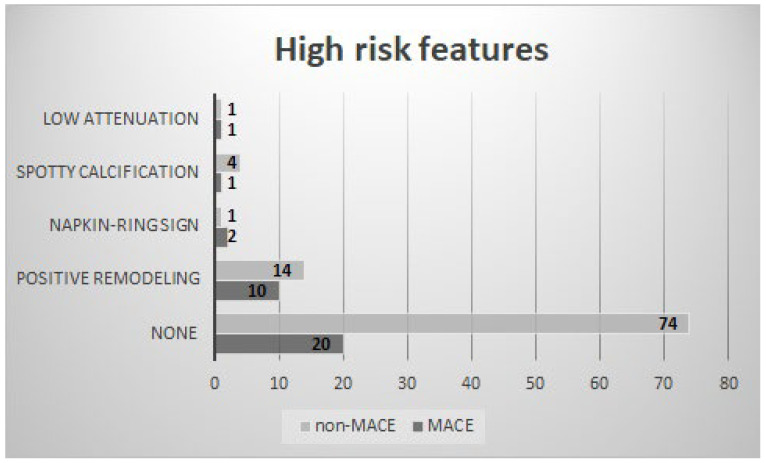
High-risk plaque features distribution among MACE and non-MACE groups. Values represent absolute counts (*n*).

High-risk plaque (HRP) features were evaluated in every intermediate coronary lesion and compared between the MACE and non-MACE groups. There were significantly more HRP in the MACE group in comparison to the non-MACE group (*n* = 10 (29.4%) and *n* = 14 (14.9%), respectively, *p* = 0.024). The most common in both groups appeared to be positive remodeling (Figure 2). Kaplan–Meier survival analysis demonstrated a non-significant trend toward poorer outcomes in patients with HRP features (Log-rank χ^2^ = 3.60, *p* = 0.058). In the Cox proportional hazards regression analysis, the presence of HRP features was associated with a significantly increased risk of MACE (HR = 2.29, *p* = 0.018). Patients with HRP features had approximately a 2.3 times higher event risk during the FU period compared to those without these features.

#### 3.2.2. Semi-Automated Analysis

Semi-automated quantitative target plaque analysis was performed on every intermediate lesion and results compared between the MACE and non-MACE groups.

On a lesion basis, only CA lumen volume (mm^3^) showed to be significantly lower in the MACE group (20.13 (12.08; 34.01) in comparison to the non-MACE group—30.12 (17.91; 49.02); *p* = 0.031). Other analyzed parameters did not differ significantly between the two groups (Table 3).

Obstruction site analysis showed significant differences between two groups: target CA sites in the MACE group had lower lumen area (3.21 (2.46; 3.92) mm^2^, non-MACE 3.99 (3.03; 4.91) mm^2^, *p* = 0.005), mean lumen diameter (2.02 (1.75; 2.14) mm, non-MACE 2.22 (1.92; 2.50) mm, *p* = 0.007), minimal lumen diameter (1.73 (1.61; 1.90) mm, non-MACE 1.93 (1.67; 2.23) mm, *p* = 0.015) in comparison to the non-MACE group. Analyzed vessel wall parameters were also significantly lower in the MACE group: vessel wall area (MACE—9.29 ± 4.27 mm^2^, non-MACE 12.26 ± 7.03 mm^2^, *p* = 0.036) and vessel wall diameter (MACE—3.52 (2.64; 3.90) mm, non-MACE 3.67 (3.15; 4.31) mm, *p* = 0.044). However, derivative parameters, such as the eccentricity index, plaque burden and vessel wall remodeling index did not differ significantly between the two groups. Lumen area stenosis was equally distributed between groups. The analysis of plaque consistency revealed a significantly bigger necrotic core area (in absolute measurements—mm^2^ (*p* = 0.044) and percentages (*p* = 0.017)) in the MACE group plaques (Table 4).

### 3.3. MACE Prediction

In univariate logistic regression analysis, lesion-area plaque composition and morphology variables were not significantly associated with the outcome (Table 5). The association of obstruction site variables in particular was, however, marked. The decreases in lumen area (OR 0.673, 95% CI 0.464–0.976; *p* = 0.037) and smaller lumen mean diameter (OR 0.255, 95% CI 0.074–0.878; *p* = 0.030) were significantly associated with MACE. Vessel wall area was also found to be negatively related to MACE (OR 0.880, 95% CI 0.787–0.984; *p* = 0.025). No other lesion-morphology variables, such as plaque burden, lumen diameter stenosis or plaque components, such as necrotic core area, had significant univariate associations. In multivariate logistic regression, the combined model including lumen area, mean lumen diameter, vessel wall area, and necrotic core area was statistically significant (Omnibus Test of Model Coefficients χ^2^ = 10.369, df = 4, *p* = 0.035), indicating an overall improvement in model fit. However, the predictive performance of the model was characterized by high specificity (92.5%) but low sensitivity (36.4%), reflecting a limited ability to identify all patients who subsequently experienced MACE. Importantly, none of the individual variables remained independently associated with MACE after adjustment, including lumen area (Exp(B) = 1.528, *p* = 0.730), mean lumen diameter (Exp(B) = 0.072, *p* = 0.531), and vessel wall area (Exp(B) = 0.922, *p* = 0.251). Necrotic core area had the highest point estimate and the lowest *p* value among the variables (Exp(B) = 2.819, *p* = 0.084), but did not reach conventional statistical significance. These findings indicate that while combined lesion characteristics may improve overall model performance, no single parameter provided sufficient standalone predictive value for MACE in this cohort.

## 4. Discussion

For the six-year FU study of 133 patients with intermediate (50–69%) stenoses identified by CCTA and without prior clinical manifestation of CAD, we observed that (1) the overall atherosclerotic burden (estimated as Agatston calcium score, CT-Leaman, and SIS) did not vary significantly between MACE and non-MACE patients; (2) HRP features in CCTA were more frequently found in the MACE group; (3) among semi-automated quantitative plaque parameters, lumen and vessel wall parameters were higher in the non-MACE group; (4) the plaque had a bigger necrotic core area in the MACE group; (5) and smaller vessel lumen area, mean lumen diameter and vessel wall area at the obstruction site had a significant difference between MACE versus non-MACE groups, although in multivariable modeling none of them showed significance alone (only necrotic core showed a trend); interestingly, when combined with the necrotic core area, the overall model showed promising statistical significance for predicting MACE.

Our observation that traditional global atherosclerotic burden evaluation did not discriminate outcomes is consistent with the concept that plaque morphology and composition may offer incremental prognostic value beyond regular stenosis severity or calcified burden evaluation. Earlier work has demonstrated that a significant proportion of acute coronary events arise from lesions with only modest luminal narrowing on conventional angiography, underscoring the limitation of stenosis-centric risk stratification [19].

In line with giving credits to plaque composition, CCTA studies have increasingly highlighted that HRP features such as low-attenuation plaque (LAP), napkin-ring sign, positive remodeling, and spotty calcification are associated with adverse events. For example, in the multicenter SCOT-HEART Trial cohort, low-attenuation non-calcified plaque predicted MI [20]. More recently, Kinoshita et al. correlated HRP features on CCTA with optical coherence tomography and further validated their prognostic importance [21]. JACC published “The 2025 consensus statement”, emphasizing that although HRP features show strong overall predictive performance, their translation into treatment recommendations remains limited [22]. Our findings of a significant association (HR ≈ 2.3) for HRP presence is thus consistent with this growing literature and supports the value of characterizing plaque beyond stenosis alone in intermediate lesions [23,24].

Qualitative analysis of plaques in intermediate coronary lesions using semi-automated techniques in this study illustrated that patients suffering from MACE, whose lumen dimensions were considerably decreased (lower values for CA lumen volume, lumen area, and mean and minimum lumen diameter). This study also showed decreased vessel wall area and diameter in these patients compared with patients without MACE. In contrast, conventional derivative analysis of the eccentricity index, plaque burden, remodeling index, and visually observed lumen stenosis did not differ between the two patient groups. This discrepancy may reflect differences in patient population (intermediate stenosis only, stable angina and no prior CAD), imaging software/analysis technique, sample size and event rates, or follow-up duration. MACE-associated plaque showed a markedly larger necrotic core component. These findings are in line with recent evidence from quantitative CCTA or intravascular imaging that plaque composition, in particular lipid-rich or necrotic core components, provides more prognostic information than stenosis severity by itself. Research by Lu et al. observed that the quantitative evaluation of the plaque load rich in lipids can predict occurrences associated with culprit lesions without revascularization independent of the severity of stenosis in patients with non-ST elevation acute coronary syndrome [25]. Similarly, intravascular ultrasound assessments have shown that the percentage and area of necrotic core are independent predictors of recurrent events following percutaneous coronary intervention [26]. Recent reviews and meta-analyses show that features such as low attenuation and the presence of necrotic core consistently result in a superior stratification for MACE risk versus diameter stenosis and overall plaque burden [27,28,29,30]. As a result, our results build on the growing evidence suggesting that among the heterogeneous range of intermediate lesions, a detailed quantitative measurement of lumen morphology and plaque pattern, rather than just stenosis grading, provides additional prognostic information and could lead to further risk stratification in clinical practice.

Among our cohort, univariate logistic regression analysis determined that only those parameters, demonstrating lumen geometry and vessel dimensions—specifically lumen area, mean lumen diameter, and vessel wall area—were significantly associated with MACE. Plaque composition and classical morphologic variables such as plaque burden and necrotic core area were not significant. Although our multivariate model of lumen area, mean lumen diameter, vessel wall area, and necrotic core area was significant overall, none of the individual predictors reached independent significance and only necrotic core area showed a trend alone. These results support the conclusion by implying that subtle changes regarding lumen geometry and vessel architecture, rather than level of plaque burden or classical HRP composition, are important predictors of future clinical events in the intermediate lesion population [31]. This is consistent with new evidence: a prospective intravascular-imaging analysis, which showed plaque–lumen geometric heterogeneity with measurements of curvature, lumen irregularity, aspect ratio and roughness were significant predictors of MACE, independent of conventional metrics for plaque composition and stenosis [32]. Additionally, on recent consensus recommendations by imaging experts, it was argued that comprehensive plaque quantification with regard to lumen and vessel geometry, plaque location and composition is needed to achieve accurate stratification of risk [33]. This, once again, makes our data part of a growing literature supporting a paradigm shift which states that for intermediate lesions and also non-obstructive lesions, a detailed quantitative analysis of the geometrical aspects of lumen and vessel dimensions (not only plaque burden or composition) can guide accurate prognostic assessment. Evaluating these metrics in routine imaging assessments may enhance the identification of HRP that would otherwise be overlooked with more traditional stenosis-specific criteria.

Thus, our data suggest that in a stable intermediate stenosis cohort, local luminal compromise and vessel wall dimensions at the lesion level may have greater prognostic relevance than global plaque burden alone. Although necrotic core area has been previously associated with MACE in meta-analyses [30], it did not reach independent statistical significance in the present multivariable analysis. However, the observed statistically significant differences in univariate analyses and the inclusion of the necrotic core area in our combined predictive model demonstrate possible incremental prognostic benefit and highlight an important direction for future research. These findings support further investigation of integrated, multi-parametric imaging approaches to improve risk stratification in patients with intermediate coronary lesions.

## 5. Conclusions

This study demonstrates that in patients with intermediate CA stenosis, lesion-specific quantitative CCTA derived parameters provide additional prognostic value beyond traditional stenosis severity and global plaque burden assessment. In particular, reduced luminal dimensions and vessel wall geometry at the obstruction site, together with the presence of visually defined high-risk plaque features, were associated with an increased long-term risk of MACE, whereas calcium score and total plaque burden alone did not discriminate outcomes.

Furthermore, lesions associated with adverse events exhibited larger necrotic core areas, supporting the concept that plaque composition contributes to plaque vulnerability and long-term risk stratification. However, no single quantitative parameter independently predicted outcomes, highlighting the importance of an integrated multi-parametric assessment approach.

From a clinical perspective, these findings suggest that incorporating detailed quantitative plaque and lumen morphology analysis into routine CCTA interpretation may improve the identification of higher-risk patients with intermediate lesions who could benefit from intensified preventive strategies and closer FU. At the same time, these imaging features should be interpreted as prognostic markers rather than direct indications for revascularization.

Overall, our results support a shift from stenosis centered evaluation toward a comprehensive, lesion-specific assessment combining stenosis severity, plaque morphology, composition, and local luminal geometry to refine long-term risk stratification in patients with intermediate CAD.

## 6. Limitations

This study has several limitations. Although the study was conducted prospectively, it was performed at a single center with a relatively limited sample size, which may have reduced statistical power and limited the implication of the findings. The study population consisted mainly of patients with low to intermediate CAD risk profiles, which may not fully represent the entire clinical spectrum of CAD and may limit application to higher-risk populations. Furthermore, longitudinal assessment of cardiovascular risk factor control and medication adherence during the FU period was not performed and may have influenced long-term outcomes. In addition, functional ischemia assessment was not included in the study protocol.

Finally, the most frequent component of MACE was target lesion revascularization. Although inclusion of revascularization in the composite endpoint may introduce potential treatment-related bias, in the present study decisions regarding invasive angiography and subsequent revascularization were based on an integrated clinical assessment, including patient symptoms, clinical presentation, ECG findings, and functional testing when available, in addition to CCTA results. Therefore, revascularization decisions were not driven by CCTA findings alone, which reduces the likelihood of circular outcome dependency.

## Figures and Tables

**Table 1:** Patients’ demographics.

Demographic Parameter	Total *n* = 128 (100%)	MACE	Non-MACE	*p* Value
		*n* = 34 (26.6%)	*n* = 94 (73.4%)	
Age, years	66.3 (10.0)	67.8 (10.4)	66.4 (9.4)	0.356
Male (%)	59 (46.1)	17 (50.0)	42 (44.7)	0.594
BMI (kg/m^2^)	30.1 (26.5; 32.8)	29.4 (25.0; 31.9)	30.4 (26.8; 33.4)	0.186
Hypertension (%)	109 (84.4)	32 (94.1)	77 (81.9)	0.086
Diabetes mellitus (%)	21 (16.4)	6 (17.6)	15 (16.0)	0.547
Smoking (%)	24 (18.8)	6 (17.6)	18 (19.1)	0.848
Dyslipidemia (%)	95 (74.2)	27 (79.4)	68 (72.3)	0.419
Total cholesterol, mmol/L	6.1 (5.1; 6.8)	6.0 (4.8; 7.4)	6.1 (5.1; 6.8)	0.919
HDL, mmol/L	1.4 (1.2; 1.5)	1.3 (1.2; 1.4)	1.4 (1.2; 1.5)	0.257
LDL, mmol/L	4.0 (3.1; 4.6)	4.0 (2.9; 5.0)	4.1 (3.1; 4.6)	0.721
Triglyceride, mmol/L	1.3 (1.0; 2.0)	1.4 (1.0; 2.0)	1.3 (1.0; 2.0)	0.871
Non-HDL, mmol/L	4.7 (3.7; 5.5)	4.8 (3.7; 6.1)	4.7 (3.7; 5.4)	0.670
Creatinine, µmol/L	78.0 (68.0; 90.0)	81.7 (71.8; 94.0)	76.8 (67.2; 87.0)	0.086
Statin use (%)	90 (70.3)	28 (82.4)	62 (66.0)	0.073

BMI—body mass index; HDL—high density lipoprotein; LDL—low density lipoprotein.

**Table 2:** Coronary atherosclerotic burden scores.

Score System	Total	MACE	Non-MACE	*p* Value
Agatston Score	100.0 (28.0; 266.0)	130.5 (50.0; 363.0)	93.0 (23.5; 248.5)	0.136
CT-adapted Leaman Score	31.5 (22.5; 43.5)	30.8 (20.3; 49.6)	31.5 (24.0; 40.5)	0.651
SIS	6.0 (4.0; 8.0)	6.5 (4.0; 9.3)	6.0 (3.0; 7.5)	0.191

CT—computed tomography; SIS—segment involvement score. Statistics layout: mean (SD) for normally distributed data; median (Q1; Q3) for non-normally distributed data.

**Table 3:** Quantitative CCTA analysis of target lesion: lesion-level parameters.

	MACE *n* = 34 (26.6%)	Non-MACE *n* = 94 (73.4%)	*p* Value
Parameter			
Lesion length, mm	5.20 (2.97; 8.7)	5.41 (3.67; 9.01)	0.330
Vessel volume, mm^3^	53.38 (21.21; 81.13)	59.44 (37.82; 98.62)	0.156
Lumen volume, mm^3^	20.13 (12.08; 34.01)	30.12 (17.91; 49.02)	0.031
Plaque volume, mm^3^	27.38 (8.06; 53.15)	30.89 (15.93; 49.41)	0.625
Mean PB, %	47.98 (12.71)	44.26 (13.00)	0.152
Maximal plaque thickness, mm	1.71 (0.72)	1.72 (0.70)	0.897
TAG mean, HU/mm	−4.99 (15.42)	−4.83 (17.73)	0.532
TAG patch mean, HU/mm	−8.20 (−18.00; 4.11)	−2.73 (−12.83; 7.83)	0.241
Fibrous volume, mm^3^	13.33 (5.64; 21.07)	14.45 (7.41; 26.73)	0.340
Percent fibrous volume, %	50.06 (18.73)	53.02 (21.41)	0.434
Fibrous fatty volume, mm^3^	2.00 (1.18; 3.74)	2.98 (1.59; 5.26)	0.120
Percent fibrous fatty volume, %	13.00 (4.50; 23.50)	13.67 (6.00; 23.41)	0.848
Necrotic core volume, mm^3^	0.62 (0.29; 4.64)	1.06 (0.37; 3.08)	0.452
Percent necrotic core volume, %	6.33 (1.00; 20.25)	4.00 (1.00; 14.25)	0.727
Dense calcium volume, mm^3^	3.38 (0.01; 19.35)	2.08 (0.01; 16.49)	0.661
Percent dense calcium volume, %	11.50 (0.00; 39.00)	11.50 (0.00; 31.50)	0.707

TAG—transluminal volume gradient; HU—Hounsfield units, PB—plaque burden. Statistics layout: mean (SD) for normally distributed data; median (Q1; Q3) for non-normally distributed data.

**Table 4:** Quantitative CCTA analysis of target lesion: obstruction-site parameters.

	MACE *n* = 34 (26.6%)	Non-MACE *n* = 94 (73.4%)	*p* Value
Parameter			
Lumen area, mm^2^	3.21 (2.46; 3.92)	3.99 (3.03; 4.91)	0.005
Lumen mean diameter, mm	2.02 (1.75; 2.14)	2.22 (1.92; 2.50)	0.007
Lumen minimal diameter, mm	1.73 (1.61; 1.90)	1.93 (1.67; 2.23)	0.015
Vessel wall area, mm^2^	9.29 (4.27)	12.26 (7.03)	0.036
Vessel wall diameter, mm	3.52 (2.64; 3.90)	3.67 (3.15; 4.31)	0.044
Eccentricity index	0.89 (0.80; 0.95)	0.91 (0.81; 0.96)	0.545
PB, %	60.26 (14.98)	56.87 (15.72)	0.278
Minimal plaque thickness, mm	0.11 (0.07; 0.20)	0.12 (0.05; 0.31)	0.656
Maximal plaque thickness, mm	1.43 (0.95; 2.09)	1.62 (1.06; 2.13)	0.299
Vessel wall remodeling index	0.81 (0.17)	0.86 (0.25)	0.296
Lumen area stenosis, %	53.70 (15.94)	50.74 (13.40)	0.297
Lumen diameter stenosis, %	33.02 (12.23)	30.71 (9.26)	0.257
Fibrous area, mm^2^	2.65 (1.24; 4.02)	3.03 (1.90; 4.21)	0.274
Percent fibrous area, %	45.73 (21.43)	50.64 (23.40)	0.292
Fibrous fatty area, mm^2^	0.48 (0.35; 0.93)	0.56 (0.31; 0.93)	0.957
Percent fibrous fatty area, %	11.92 (5.19; 27.50)	11.00 (4.00; 25.00)	0.467

PB—plaque burden. Statistics layout: mean (SD) for normally distributed data; median (Q1; Q3) for non-normally distributed data.

**Table 5:** Prognostic value.

	Univariate Logistic Regression Model		Multivariate Logistic Regression Model	
	OR (95% CI)	*p* Value	aOR (95% CI)	*p* Value
Lesion area:				
Lesion length, mm	0.949 (0.859–1.048)	0.302	–	–
Vessel volume, mm^3^	0.994 (0.985–1.003)	0.168	–	–
Lumen volume, mm^3^	0.983 (0.965–1.002)	0.084	–	–
Plaque volume, mm^3^	0.995 (0.981–1.010)	0.510	–	–
Mean PB, %	1.022 (0.992–1.054)	0.153	–	–
Maximal plaque thickness, mm	0.967 (0.550–1.698)	0.906	–	–
TAG mean, HU/mm	0.999 (0.975–1.024)	0.963	–	–
TAG patch mean, HU/mm	0.998 (0.978–1.018)	0.828	–	–
Fibrous volume, mm^3^	0.980 (0.950–1.011)	0.201	–	–
Percent fibrous volume, %	0.993 (0.974–1.012)	0.473	–	–
Fibrous fatty volume, mm^3^	0.985 (0.899–1.078)	0.736	–	–
Percent fibrous fatty volume, %	1.003 (0.975–1.033)	0.821	–	–
Necrotic core volume, mm^3^	1.020 (0.950–1.096)	0.583	–	–
Percent necrotic core volume, %	1.007 (0.981–1.033)	0.603	–	–
Dense calcium volume, mm^3^	1.000 (0.978–1.022)	1.000	–	–
Percent dense calcium volume, %	1.004 (0.985–1.024)	0.675	–	–
Area of obstruction:				
Lumen area, mm^2^	0.673 (0.464–0.976)	0.037	1.528 (0.138–16.924)	0.730
Lumen mean diameter, mm^2^	0.255 (0.074–0.878)	0.030	0.072 (0.000–272.353)	0.531
Lumen minimal diameter, mm	0.769 (0.335–1.764)	0.535	–	–
Vessel wall area, mm^2^	0.880 (0.787–0.984)	0.025	0.922 (0.802–1.060)	0.251
Vessel wall diameter, mm	1.037 (0.962–1.118)	0.340	–	–
Eccentricity index	0.356 (0.006–20.014)	0.616	–	–
PB, %	1.014 (0.989–1.041)	0.277	–	–
Minimal plaque thickness, mm	0.650 (0.133–3.177)	0.594	–	–
Maximal plaque thickness, mm	0.684 (0.358–1.306)	0.250	–	_
Vessel wall remodeling index	0.340 (0.045–2.559)	0.295	–	–
Lumen area stenosis, %	1.015 (0.987–1.045)	0.295	–	–
Lumen diameter stenosis, %	1.023 (0.984–1.063)	0.257	–	–
Fibrous area, mm^2^	0.883 (0.705–1.106)	0.279	–	–
Percent fibrous area, %	0.990 (0.973–1.008)	0.291	–	–
Fibrous fatty area, mm^2^	1.277 (0.704–2.317)	0.422	–	–
Percent fibrous fatty area, %	1.006 (0.981–1.032)	0.642	–	–
Necrotic core area, mm^2^	0.992 (0.838–1.174)	0.927	2.819 (0.871–9.130)	0.084
Percent necrotic core area, %	1.021 (0.999–1.043)	0.061	–	–
Dense calcium area, mm^2^	0.961 (0.861–1.073)	0.479	–	–
Percent dense calcium area, %	0.995 (0.978–1.012)	0.567	–	–

TAG—transluminal volume gradient; HU—Hounsfield units, PB—plaque burden. Statistics layout: mean (SD) for normally distributed data; median (Q1; Q3) for non-normally distributed data.

## Data Availability

The original contributions presented in this study are included in the article. Further inquiries can be directed to the corresponding author(s).

## Abbreviations

BMI—body mass index; CA—coronary artery; CT—computed tomography; CAD—coronary artery disease; CAD-RADS—Coronary Artery Disease Reporting and Data System; CI—confidence interval; CCTA—cardiac computed tomography angiography; ECG—electrocardiogram; ESC—European Society of Cardiology; FU—follow-up; HDL—high density lipoprotein; HR—heart rate; HRP—high-risk plaque; HU—Hounsfield units; ICC—intra-class correlation coefficient; IQR—interquartile ranges; IVUS—intravascular ultrasound; LAD—left anterior descending artery; LCx—left circumflex coronary artery; LVEF—left ventricular ejection fraction; LDL—low density lipoprotein; MACE—major adverse cardiovascular events; MI—myocardial infarction; PACS—picture archiving and communication system; PB—plaque burden; RCA—right coronary artery; RI—remodeling index; SD—standard deviation; TAG—transluminal volume gradient; χ^2^—Chi-square.
